# What Does Evolution Do with a Spare Set of Genes?

**DOI:** 10.1371/journal.pbio.0040132

**Published:** 2006-04-04

**Authors:** Mary Hoff

A hundred million years ago, a molecular twist of fate endowed an ancestor of today's baker's yeast *(Saccharomyces cerevisiae)* with an extra copy of every gene it owned—the equivalent of a factory one day finding double the number of workers reporting for duty. What did the yeast and the forces of evolution do with this treasure trove of potential? Did the extra gene-workers simply double the output? Did the original crew and the duplicates divvy up the ancestral functions? Or did they take on new tasks? That's what Gavin Conant and Kenneth Wolfe sought to find out in their study of the networks of interactions among genes and other cellular components that emerged in the wake of that landmark event.

Some of the genes from the original doubling disappeared completely from the *S. cerevisiae* genome in the intervening millennia. But previous research had identified 551 duplicate gene (paralog) pairs that remain. To explore their fate, the authors used information about known co-expression from other *S. cerevisiae* studies along with an algorithm they developed on these genes pairs, and they identified 19 networks made up of paralogs divided such that there are many interactions within each network but few between the two paired networks. They then set out to explore the extent to which the networks composed of the two sets of paralogs differed from each other—a measure of the degree to which they had diverged evolutionarily, and so taken on separate functions, over time.

The first test looked at symmetry between the networks formed by the two sets of paralogs. The researchers found that for many of the network pairs, one set of paralogs had significantly more interactions than the other. The networks also had more redundancy—multiple interactions between two pairs of paralogs—than would be found in randomly grouped networks. These findings suggest substantial but incomplete divergence since the original gene-duplicating event.

Second, the authors explored the extent to which the 19 networks they had identified showed evidence of functional significance. To do so, they split the 551 paralog pairs into random networks, then recalculated network partitions for each. Eight of the networks showed significantly better clustering of gene interactions with respect to co-expression data than did the randomized networks, supporting the contention that they do in fact represent modular functional units, not just mathematical constructs. To further provide evidence of potential functionality, the researchers also analyzed whether partitions contained proteins with similar cellular localization and/or upstream regulatory sequence motifs. In the two largest of the networks with significant partitioning, protein localization and regulatory sequences were better conserved within each of the network partitions than would be expected by chance, confirming the functional correspondence seen with gene co-expression data.

To illustrate the adaptive value of network partitioning, the authors described a pair of paralogs whose protein products catalyze the last reaction in glycolysis. One encodes an enzyme induced by a compound present when glucose levels are high, while the other encodes an enzyme that works without this metabolic intermediate. As a result, the yeast can efficiently carry out the reaction in both high- and low-glucose environments.

Finally, the authors tested three mathematical models of network evolution against their observations as a way to gain insights into what actually happened to interactions among genes over the evolutionary history of the yeast. In the first model, which they called “uniform loss,” interactions were lost at random. In the second model, called the “poor-get-poorer” model, the probability of loss of an interaction between two genes was set to be inversely proportional to number of ancestral interactions retained. The third, “co-loss” model, in which the probability of an interaction loss depends on number of shared neighboring genes (the more shared, the less likely a loss) proved to provide the best approximation to which interactions actually were lost and retained over time. The strength of the third model supported the authors' speculation that the partitioned networks originally formed through the partial loss of old function rather than the development of new functions, in contrast to the common belief that increased complexity is largely the consequence of positive selection.

What *does* evolution do when handed a spare set of genes? In the case of *S. cerevisiae*, at least, it appears to have modified interactions among genes and other cellular components to produce a set of partially independent daughter networks from each single ancient network, creating in the process a division of labor that makes the most of the possibilities presented by the fortuitous duplication of the genome in the yeast's ancient past.

## 

**Figure:**
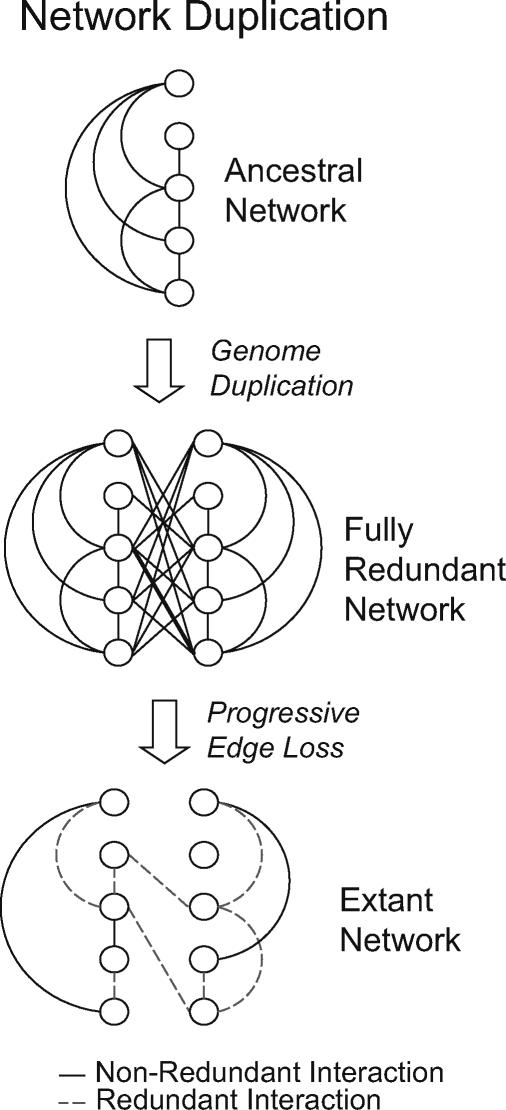
After a genome duplication event, which provides networks with many simultaneously duplicated genes (nodes), the number of nodes in the network has doubled and the number of interactions has quadrupled. The subsequent gain or loss of interactions reduces redundancy.

